# Androgenic hidradenitis suppurativa onset linked to gamma secretase inhibitor

**DOI:** 10.1016/j.jdcr.2025.10.038

**Published:** 2025-10-28

**Authors:** Hansen Tai, Khyla T. Hill, Aarthi Parvathaneni, Zahidul Islam, Steven R. Cohen

**Affiliations:** a Department of Dermatology, Weill Medical School of Cornell University, New York, New York; b SUNY Upstate Norton College of Medicine, Syracuse, New York; c Loyola Stritch School of Medicine, Maywood, Illinois; d McGovern (University of Texas, Houston) Medical School, Houston, Texas; e School of Medicine, New York Medical College, Valhalla, New York

**Keywords:** amenorrhea, androgen, desmoid tumor, gamma secretase inhibitor, hidradenitis suppurativa, menopause, nicastrin, nirogacestat, notch signaling, ovarian failure, presenilin, spironolactone, therapy

## Introduction

Hidradenitis suppurativa (HS) is a chronic inflammatory skin disorder of follicular origin, primarily affecting areas with apocrine gland-bearing skin. Recurrent painful nodules, abscesses, and sinus tracts characterize advanced HS. It leads to scarring, malodor, and intractable draining wounds, significantly impairing quality of life.^1^ Past studies have demonstrated underlying genetic etiology for HS, especially in familial HS, where genetic variation in gamma-secretase complex protein-coding genes accounts for most identified variants from genome-wide sequencing in familial and sporadic HS.^2^^,^^3^ Nirogacestat, a gamma-secretase inhibitor, was recently approved by the U.S. Food and Drug Administration for the treatment of desmoid tumors. It exerts its effects by blocking Notch signaling through the selective inhibition of gamma-secretase cleavage of Notch receptors.^4^ We present a rare and intriguing case of a patient who developed HS after 6 months of nirogacestat therapy.

To our knowledge, only 1 other case report has delineated the relationship between gamma-secretase inhibitors and the development of HS.

## Case

**Fig 1:**
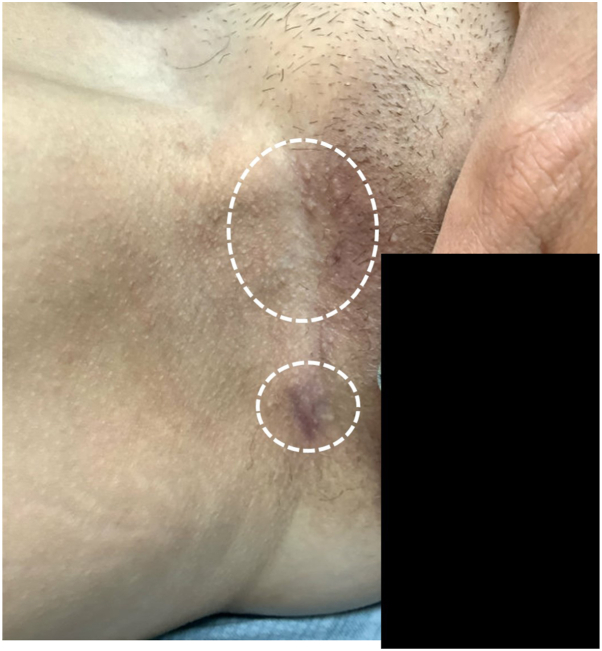
Postinflammatory nodules/scarring at the initial visit. *White circles* identify sites of postinflammatory hyperpigmentation and scarring associated with hidradenitis suppurativa.

**Fig 2:**
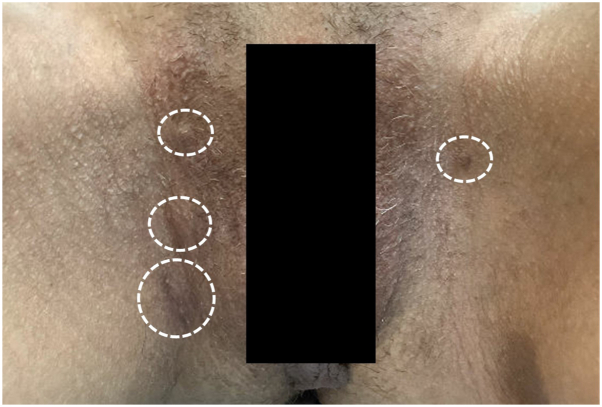
Postinflammatory hyperpigmented HS nodules involving bilateral inguinal creases at the second visit. *White circles* identify sites of postinflammatory hyperpigmentation and scarring associated with hidradenitis suppurativa. *HS*, Hidradenitis suppurativa.

A 48-year-old woman presented to our center in November 2024 with a 4-month history of HS-like lesions in her right upper inner thigh that flared monthly. She began nirogacestat 150 mg twice daily in December of 2023 to treat her right abdominal wall desmoid tumor, first identified in 2021 during her pregnancy. The tumor enlarged peri- and postpartum, and she attempted chemotherapy with Doxil (40 mg/m^2^) in September of 2023 but terminated the treatment due to infusion reactions. Nirogacestat was held for 2 months due to a severe rash that developed on January 2, 2024, requiring prednisone, hydroxyzine, and triamcinolone. Nirogacestat was restarted at 50 mg twice daily and escalated back to 150 mg twice daily as of April 8, 2024, increased by 1 tablet every 2 weeks. After 3 months, inflammatory nodules of HS appeared on the right inner thigh. Concomitant side effects included diarrhea, fatigue, amenorrhea, and daily hot flashes consistent with menopause onset. HS remained localized while flares occurred monthly. Acute lesions were refractory to intralesional triamcinolone, Augmentin, and topical therapies. At presentation to our center, HS was clinically diagnosed with 4 to 5 areas of posinflammatory hyperpigmentation and scarring observed in the right inguinal crease and upper medial thigh (Fig 1). Topical chlorhexidine wash and clindamycin gel, combined with oral spironolactone 50 mg twice daily, as well as minocycline 100 mg twice daily, led to reduced HS activity (Fig 2).

## Discussion

Notch signaling is a highly conserved pathway that regulates cell differentiation, fate, and proliferation. Gamma-secretase plays a critical role in cleaving the transmembrane domain of Notch to allow the activated Notch intracellular domain to travel to the nucleus and regulate transcriptional complexes.^5^ Mutations in gamma-secretase subunit genes such as presenilin, presenilin enhancer 2, and nicastrin result in abnormal follicular keratinization and epidermal hyperplasia.^6^ First demonstrated in a mouse model where genetic inactivation of gamma-secretase produced follicular abnormalities similar in histology to HS, all 3 genes have been implicated in familial HS, with nicastrin being the most commonly identified.^3^ A previous study during the Phase II trial of nirogacestat found 12 of the 17 patients undergoing therapy developed follicular lesions in intertriginous areas (axilla, inguinal crease, buttocks, and thigh) with resolution of these effects after cessation of treatment, prompting the authors to suggest that onset of HS does not require developmental or pubertal insufficiency in gamma-secretase, but with targeted inactivation in patients with normal skin.^7^

In the Phase III clinical trial of nirogacestat, 6 of 70 (9%) patients experienced hidradenitis as a cutaneous adverse event, while 27 of 36 (75%) female patients of childbearing age experienced ovarian dysfunction (amenorrhea, menopause, premature menopause, and ovarian failure). Laboratory findings included increased levels of follicle-stimulating hormone and decreased levels of estradiol. The median time to symptom onset was 8.9 weeks, with 20 of the 27 patients experiencing clinical resolution after a median duration of 19.1 weeks. Five patients did not experience resolution of their symptoms.^4^ The relationship between HS and endocrinologic factors has been extensively documented due to the disease's occurrence within a narrow window between puberty and onset, as well as its association with obesity and menstrual cycles; however, the exact pathophysiology remains unclear. The majority of studies have shown no alteration in major sex hormones in HS patients. Studies attempting to delineate whether increased peripheral androgen conversion, as well as increased androgen and estrogen receptor expression in HS tissue, have found no difference.^8^ Nonetheless, antiandrogen therapy has demonstrated efficacy in treating HS patients, with spironolactone and finasteride being a mainstay at our center.^9^

Our patient experienced the onset of ovarian dysfunction (amenorrhea and daily hot flashes) before the onset of her HS symptoms, potentially pointing towards an androgenic aspect in her disease onset. Similar to previous studies, her total testosterone level was normal at 17 ng/dL; however, she experienced great disease control with spironolactone monotherapy.

## Conclusion

In conclusion, we report a rare case of HS developing after initiation of nirogacestat therapy, highlighting a possible association between gamma-secretase inhibition and HS pathogenesis. Given that gamma-secretase inhibitors disrupt Notch signaling, a critical regulator of follicular development and epidermal homeostasis, clinicians should remain vigilant for cutaneous adverse events, including HS, during treatment. Additionally, the concurrent onset of ovarian dysfunction before HS in our patient suggests a potential endocrine interplay that warrants further investigation. Early identification and management, as illustrated by the efficacy of spironolactone therapy in our patient, may mitigate disease burden and improve patient outcomes. Additional studies are needed to elucidate the precise mechanisms linking gamma-secretase inhibition, endocrinologic changes, and HS pathogenesis.

## Conflicts of interest

None disclosed.

## References

[1] Chu Y.L., Yu S. (2024). Hidradenitis suppurativa: an understanding of genetic factors and treatment. Biomedicines.

[2] Pace N.P., Mintoff D., Borg I. (2022). The genomic architecture of hidradenitis suppurativa-A systematic review. Front Genet.

[3] Wang B., Yang W., Wen W. (2010). Gamma-secretase gene mutations in familial acne inversa. Science.

[4] Gounder M., Ratan R., Alcindor T. (2023). Nirogacestat, a γ-secretase inhibitor for desmoid tumors. N Engl J Med.

[5] Kopan R. (2012). Notch signaling. Cold Spring Harb Perspect Biol.

[6] Jfri A.H., O'Brien E.A., Litvinov I.V., Alavi A., Netchiporouk E. (2019). Hidradenitis suppurativa: comprehensive review of predisposing genetic mutations and changes. J Cutan Med Surg.

[7] O'Sullivan Coyne G., Woodring T.S., Lee C.C.R., Chen A.P., Kong H.H. (2018). Hidradenitis suppurativa-like lesions associated with pharmacologic inhibition of gamma-secretase. J Invest Dermatol.

[8] Karagiannidis I., Nikolakis G., Zouboulis C.C. (2016). Endocrinologic aspects of hidradenitis suppurativa. Dermatol Clin.

[9] Babbush K.M., Andriano T.M., Cohen S.R. (2022). Antiandrogen therapy in hidradenitis suppurativa: finasteride for females. Clin Exp Dermatol.

